# Phenolic Compounds Accumulation and Cell Death Degree Induced by Fusaric Acid in Agroforestry Hosts Plants of *Fusarium* Species

**DOI:** 10.3390/jof11100745

**Published:** 2025-10-17

**Authors:** Angélica Gutiérrez-Sánchez, Javier Plasencia, Juan L. Monribot-Villanueva, Benjamín Rodríguez-Haas, Eliel Ruiz-May, José A. Guerrero-Analco, Diana Sánchez-Rangel

**Affiliations:** 1 Laboratorios de Fitopatología y Biología Molecular, Red de Estudios Moleculares Avanzados, Instituto de Ecología, A. C., Xalapa 91073, Veracruz, Mexico; angelica.4255@gmail.com (A.G.-S.); benjamin.rodriguez@inecol.mx (B.R.-H.); 2 Laboratorio de Química de Productos Naturales, Red de Estudios Moleculares Avanzados, Instituto de Ecología, A. C., Xalapa 91073, Veracruz, Mexico; juan.monribot@inecol.mx; 3 Departamento de Bioquímica, Facultad de Química, Universidad Nacional Autónoma de México, Ciudad de México 04510, Mexico; javierp@unam.mx; 4 Independent Researcher, Coatepec 91540, Veracruz, Mexico; pelecas40@gmail.com; 5 Investigadora por México—SECIHTI, Red de Estudios Moleculares Avanzados, Instituto de Ecología, A. C., Xalapa 91073, Veracruz, Mexico

**Keywords:** fusaric acid, scopoletin, Fusarium dieback, phenolic compounds, *Fusarium*, coumarins

## Abstract

The genus *Fusarium* comprises multiple species recognized as plant pathogens in both annual and perennial crops. Some phytopathogenic species of this genus can be transmitted by insect vectors, which introduce them into woody plant species of ecological and agroeconomic importance. Among these species, *Fusarium kuroshium* stands out, but studies are limited because it is a quarantine pathogen that requires special biosafety measures for its culture. This fungus produces fusaric acid (FA), a virulence factor that is widespread in *Fusarium* spp. To gain insight into the role of this phytotoxin in virulence, we exposed leaves of four woody host species (*Liquidambar styraciflua*, *Persea americana*, *Citrus sinensis*, and *Populus nigra*) of *F. kuroshium* to FA in vitro. The plant tissue exhibited varying degrees of cell death and physiological alterations, including a reduction in biomass, generation of reactive oxygen species (ROS), elevated electrolyte leakage, and loss of photosynthetic pigments. A chemical analysis demonstrated that the flavonoid and isoflavonoid pathways, in addition to linoleic and linolenic acid metabolism, were markedly affected by FA. Following the quantification of phenolic compounds in leaves, 11 metabolites were identified whose concentrations increased in response to FA stress. The findings of this study indicate that phenolic compounds play a significant role in the response to FA stress. Particularly, scopoletin has a protective effect on leaves of *Liquidambar styraciflua*.

## 1. Introduction

Fusaric acid (FA) is a wilt toxin that has been identified as a virulence factor in several species of filamentous fungi of the *Fusarium* genus, mainly because of its important role in early stages of the plant–pathogen interaction, as it diffuses ahead of the fungal invasion [1]. Unlike other toxins such as fumonisins or trichothecenes, which are taxonomically restricted to certain species, FA is biosynthesized by almost all *Fusarium* species and its production by pathogenic strains has been related to their level of virulence [2,3]. Despite its wide distribution in the genus, its production and concentration depend on several environmental factors, including an alkaline extracellular pH, low iron availability, deficient nitrogen sources, and signaling by cell wall integrity mitogen-activated protein kinase [4]. Fusaric acid, like other toxins, is secreted by the fungi into the vessels and transported to the leaves, where it affects various physiological processes [1]. The initial signs of disease are apparent at the tissue of the leaves, such as a decrease in photosynthetic pigments, browning, and necrosis [5]. In tomato cells, FA causes cell death accompanied by an increase in extracellular pH and superoxide ion (O_2_^−^) production, along with a rise in intracellular activity of antioxidant enzymes [6]. Chemically, FA can function as a weak acid, which facilitates diffusion across membranes in its non-ionized form. Once the toxin is internalized, its ionization results in the release of H^+^ that acidifies the cytosol, ultimately leading to hyperpolarization of the plant cell [7,8]. The activity of FA extends beyond its primary mechanism of action of altering membrane potential, as it is capable of interacting with cellular organelles such as the cell membrane or mitochondria [9]. Recently, a model was proposed where FA stimulates H_2_O_2_ production, enhancing the permeability of the mitochondrial membrane and subsequent cytochrome C release, which results in the activation of caspase-like proteins that induce programmed cell death [9,10]. Moreover, FA has the ability to chelate essential plant metal ions, providing another mechanism of phytotoxicity [11]. Most studies on FA have been carried out on model organisms, including *Arabidopsis thaliana* or *Egeria densa*, as well as on agriculturally significant crops like banana, cucumber, maize, and tomato [1,5,8,12,13,14]. However, little is known on the effects of this virulence factor on woody plants. Its relevance is highlighted due to emergent phytopathogenic fungi that can infect woody plant species of both forestry and economic importance. For example, the fungus *Fusarium kuroshium*, vectorized by the beetle *Euwallacea kuroshio*, which was first detected in North America in 2012 in avocado trees [15]. In optimal environmental conditions, the fungus colonizes and infects the host plant, causing Fusarium dieback (FD) disease [16]. This species produces FA, which possesses phytotoxic effects on *Persea americana* leaves, characterized by browning and wilting at a concentration of 2.5 mM [17]. Thus, FA might play a role in FD symptom development in woody host species. Because *F. kuroshium* is a quarantine pathogen that requires BSL3 facilities for its culture and manipulation, we set out to study the effects of FA on woody plant species that are potential hosts of this fungus. There are several reports about the effect of FA on the physiology of model plants but none of them were susceptible to FD, so the objective of this work was to explore the biochemical and metabolic changes caused by this toxin in selected woody plants of agricultural and forestry interest that are potential hosts of *F. kuroshium* and other *Fusarium* species.

## 2. Materials and Methods

### 2.1. Fusaric Acid Leaf Exposure Bioassay

To investigate the effect of FA on leaf tissue, four FD host plants were included in the present study from those reported by [18]. The selected trees species were *Liquidambar styraciflua* (sweetgum), *Persea americana* (avocado), *Citrus sinensis* (orange), and *Populus nigra* (poplar). The individuals were grown in a greenhouse under controlled conditions with a relative humidity range of 60–80%, and a maximum and minimum temperature of 25 °C and 19 °C, respectively. The phytotoxicity bioassay was carried out as described by [17] with minor modifications. Given the observed variations in leaf size among the four species under investigation, leaf discs of 13 mm diameter were employed to standardize the surface exposed to the toxin. The discs were cut and randomly placed in a 6-well plate, where each well contained 5 mL of FA commercial standard (Sigma Aldrich, St. Louis, MO, USA, F6513) at different concentrations (0, 0.1, 0.5, 1, 2.5, 5, and 10 mM) in sterile distilled water (dH_2_O). Ten leaf discs were placed in each well and sterile dH_2_O at pH 5.00 was used as a negative control. The bioassay with all treatments was incubated in a growth chamber (Percival Scientific AR-41L3, Perry, IA, USA) at a temperature of 22 ± 1 °C, relative humidity of 65%, and 16:8 h light/dark photoperiod. After 72 h of exposure to FA, a photographic record (SONY, DSC-HX60V, San Diego, CA, USA) of the damage in fresh tissue was recorded. Leaves from each treatment were collected for measurement of parameters related to cell damage and cell death. The evaluation of the protective effect of scopoletin and luteolin against FA-generated cell death was performed exclusively on *L. styraciflua* leaves. For each treatment, 60 leaf discs were exposed to luteolin or scopoletin at 1, 10, and 100 mM for 24 h. Thereafter, the discs were transferred to a 5 mL solution of FA (5 mM) for 72 h. Leaf discs exposed to 10 mM luteolin or scopoletin were utilized as negative controls, while leaf discs exposed exclusively to 5 mM FA were employed as positive controls, all in sterile dH_2_O.

### 2.2. Assessment of Biochemical Parameters in Leaves Under FA Exposure

#### 2.2.1. Evans Blue Staining for Cell Death Measurement

The procedure for Evans blue staining was carried out as was reported by [19] with minimal modifications. We used 250 mg of Evans blue stain (E2129, Sigma Aldrich, St. Louis, MO, USA) in a solution of CaCl_2_ (1332-01, J.T. Baker, Phillipsburg, NJ, USA). Ten leaf discs were taken from each species after 72 h of exposure to FA and then 5 mL of dye was added. The discs were then placed in an orbital shaker (Thermo Scientific, MaxQ 2000, Marietta, OH, USA) at 150 rpm for one hour, after which leaves were placed in 96% ethanol (EtOH) until chlorophyll was removed. Photographic record (SONY, DSC-HX60V, San Diego, CA, USA) was taken in a stereoscopic microscope (LEICA EZ4, Heerbrugg, Switzerland). Percentages of damaged or death area were analyzed using a non-parametric Kruskal–Wallis test followed by a Dunn’s test to determine significant differences. The evaluation of scopoletin and luteolin involved the extraction and quantification of the dye, which was conducted in accordance with the methodology of [19] in a multimode microplate reader (Varioscanä LUX; Thermo Fisher, Waltham, MA, USA) at 600 nm, followed by analysis of variance (ANOVA) and Tukey test to identify significant differences in cell death.

#### 2.2.2. Hydrogen Peroxide Detection by DAB Staining

The 3,3′-diaminobenzidine (DAB) staining procedure was performed in accordance with the methodology of [20] with minimal modifications using a DAB (D12384, Sigma Aldrich, St. Louis, MO, USA) solution at 1 mg mL^−1^, pH 3.00. Ten leaf discs from each treatment were treated with 5 mL of DAB solution. The discs were shielded from light and agitated at 100 rpm for 4 h. The DAB solution was replaced with a decolorizer mixture containing ethanol–acetic acid–glycerol (3:1:1). The tissue was observed under a stereoscopic microscope (LEICA EZ4, Heerbrugg, Switzerland) to obtain a photographic record (SONY, DSC-HX60V, San Diego, CA, USA).

#### 2.2.3. Electrolyte Leakage from Cells

For electrolyte leakage measurement, we followed the protocol established by [5]. The electrical conductivity was measured using a conductivity meter (Horiba scientific, LAQUAtwin EC-33, Kyoto, Japan). Statistical analysis consisted of a Student’s *t*-test comparing the treated leaves with respect to the control leaves, for each species independently.

#### 2.2.4. Chlorophyll Quantitation

Changes in chlorophyll a and b content of leaves exposed to FA were evaluated against control plants, following the methodologies of [21] and using a spectrophotometer (Shimadzu; BioSpec-nano, Kyoto, Japan). Electrolyte leakage data and photosynthetic pigment content were analyzed by Student’s *t*-test comparing, for each species, control leaves with those exposed to 5 mM FA.

### 2.3. Metabolomics on Plant Leaves After FA Exposure

#### 2.3.1. Extraction

The leaf exposure bioassay at 5 mM FA was repeated, using a greater number of replicates (n = 60). After 72 h of exposure to the phytotoxin, the leaf discs were recovered to measure the fresh weight, then the protocol established by [22] was followed. Briefly, fresh tissue was dried in a freeze-dryer system (Labconco^®^ FreeZone1, Kansas City, MO, USA). The dry weight was recorded and methanol (MeOH; Honeywell, Chromasolv™ HPLC grade, Charlotte, NC, USA) extracts were obtained in an accelerated solvent extraction system (ASE350™, Thermo Scientific, Dionex™, Sunnyvale, CA, USA), using two cycles of extraction at 60 °C for 5 min. The extracts were dried using rotary evaporation (BÜCHI RII, Flawil, Switzerland) and then finally redissolved in MeOH (LC-MS grade, Sigma Aldrich, St. Louis, MO, USA) with formic acid at 0.1% at a final concentration of 50 mg mL^−1^. Then, the solution was filtered through PTFE membrane (0.2 μm) and placed in UPLC vials for LC-MS analysis.

#### 2.3.2. Quantification of FA in Leaf Tissue

For the quantification of FA within the leaf tissue, a calibration curve was made with the commercial standard using eight concentration points (0.5, 1, 3, 5, 7, 9, 11, and 13 µM), analyzed using high-resolution liquid chromatography equipment (UPLC; Agilent Technologies 1290 Infinity series, Santa Clara, CA, USA) coupled to a mass spectrometer with a triple quadrupole analyzer (QqQ; Agilent Technologies 6460, Santa Clara, CA, USA). Each point was injected twice, and the corresponding areas were considered to generate the calibration curve. A second-order curve regression with a coefficient of determination of 0.99 was used, and the acquisition method for MS/MS fingerprint was multiple reaction monitoring (MRM), following the conditions stablished by [17]. Data acquisition and analysis was performed with the software MassHunter Workstation version B.06.00 (Agilent Technologies, Santa Clara, CA, USA). The data were analyzed using a one-way ANOVA test to detect significant differences in intracellular FA content among the four species studied.

#### 2.3.3. Untargeted Metabolomics Analysis

Methanol extracts (5 mL) were injected into an UPLC (Waters™ Acquity Class I, Milford, MA, USA) coupled to a high-resolution mass spectrometer with a Quadrupole-Time of Flight (QToF) analyzer (LC-HRMS, Waters™ HDMi Synapt G2-Si, Milford, MA, USA) following the methodology described by [22]. Peaks intensity and retention time (Rt) for each mass/charge ratio (*m*/*z*) were acquired and processed with the software MassLynx™ version 4.1 (Waters™, Milford, MA, USA). Statistical analysis was performed in MetaboAnalyst 5.0 platform (https://www.metaboanalyst.ca/, accessed on 13 February 2023), where comparative analyses were conducted between control leaves and FA-treated leaves for each species. Putative identifications of metabolites that exhibited heightened levels in leaves exposed to FA were sought in public databases FooDB (https://foodb.ca/, accessed on 15 April 2023) and Lotus (https://lotus.naturalproducts.net/, accessed on 15 April 2023). The mass spectra for each compound were compared with the theoretical and experimental information available in databases such as Massbank (https://massbank.eu/MassBank/, accessed on 5 June 2023) or CFM-ID (https://cfmid.wishartlab.com, accessed on 5 June 2023). Two accuracy levels of identification were established: (1) the first indicates that there was a coincidence of the precursor ion and more than two fragment ions and (2) the second indicates those molecules identified by a coincidence of the precursor ion, with a maximum mass error allowed of ±5 ppm.

#### 2.3.4. Targeted Metabolomics for Phenolic Compounds

Phenolic compounds were identified and quantified using a UPLC system (Agilent, 1290, Santa Clara, CA, USA) coupled to a QqQ mass spectrometer (Agilent, 6460, Santa Clara, CA, USA) with the dynamic multiple reaction monitoring acquisition method previously described by [23]. Data were obtained with the Agilent MassHunter Workstation software (B.06.00), and the statistical analysis was carried out on the MetaboAnalyst 5.0 platform (https://www.metaboanalyst.ca/, accessed on 20 january 2024).

## 3. Results

### 3.1. Fusaric Acid Treatment Leads to Cell Death in Different Plant Species

**Figure 1:**
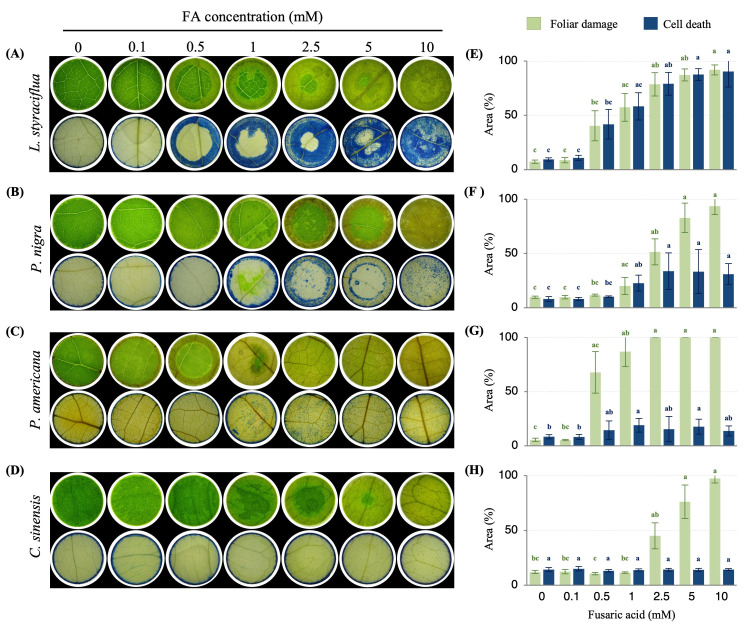
FA-induced cell damage in leaves of *L. styraciflua*, *P. nigra, P. americana,* and *C. sinensis* (**A**–**D**). Leaf damage (upper row) and Evans blue staining (lower row) (**E**–**H**). Percentage of foliar damaged area (green) and percentage of cell death area (blue) quantified using the ImageJ software version 1.54g. Different letters indicate statistical differences (*p* < 0.05) observed by Kruskal–Wallis and Dunn’s test. The photographs were taken 72 h after exposure to FA.

Phytotoxicity was evaluated by testing increasing concentrations of FA (0.1 to 10 mM) on the leaf tissue of four potential hosts of *F. kuroshium* (Figure 1). Visible lesions, characterized by a pale green to brown coloration, were observed on the leaves of all plants. *P. americana* and *L. styraciflua* were the most susceptible species, as 0.5 mM FA caused a damaged area of 67.8 and 40.4% respectively (Figure 1A,C, Figures S1 and S3). In contrast, a concentration of 2.5 mM FA generated a damaged area of 51.5% and 45.2% in *P. nigra* and *C. sinensis*, respectively (Figure 1B,D, Figures S2 and S4). At this FA concentration, *P. americana* exhibited a damaged area of 100%. The highest FA concentration tested (10 mM) caused full leaf damage in *C. sinensis*, *L. styraciflua*, and *P. nigra*.

Leaf tissue was stained with Evans blue dye to ascertain cell death and the % of the area was quantified. Only in *L. styraciflua*, the % of the area and the stained pattern were comparable to the damaged leaf area (Figure 1A and Figure S1), while the other three species displayed poor staining: *P. nigra* (Figure 1B and Figure S2) exhibited 22.7 ± 7.4% and 33.38 ± 16.8% cell death area at 1 and 2.5 mM of FA, respectively. On the other hand, *P. americana* (Figure 1C and Figure S3) exhibited a maximum of 19.07 ± 6.3% cell death area at 1 mM, and *C. sinensis* (Figure 1D and Figure S4) exhibited minimal variation around 14% cell death in all treatments.

### 3.2. FA Induces Biochemical Disturbances Within the Cells

**Figure 2:**
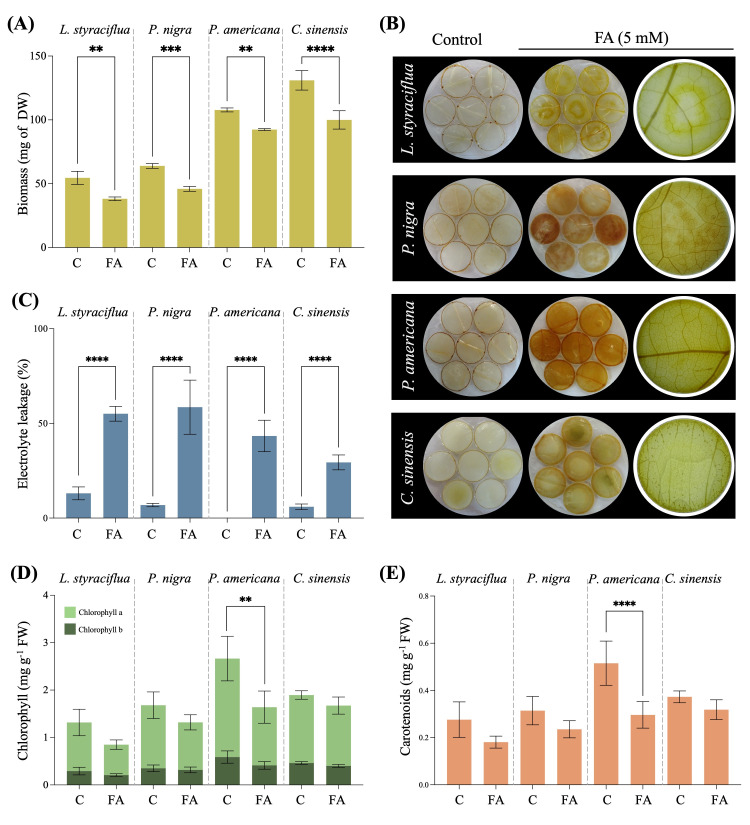
Parameters related to cell death caused by FA (5 mM). (**A**) Biomass. (**B**) Hydrogen peroxide detected in leaves by DAB staining. (**C**) Percent electrolyte leakage. (**D**) Chlorophyll a and b content. (**E**) Carotenoid content. The statistically significant difference (** *p* ≤ 0.01, *** *p* ≤ 0.001, **** *p* ≤ 0.0001) as determined by Student’s *t*-test is indicated above the bars. C: Control (dH_2_O); FA: Fusaric acid.

To further characterize the response to FA in different plant species, we measured the following parameters: leaf biomass, H_2_O_2_ production, electrical conductivity, and chlorophyll content. The leaves of all species treated with 5 mM FA exhibited a significant reduction of around 30% in biomass in relation to the mock control (Figure 2A). The greatest loss was observed in *L. styraciflua* (30%), followed by *P. nigra* (28%) and *C. sinensis* (23.6%), while the lowest biomass reduction occurred in *P. americana*, with an 14.3% loss of leaf biomass.

Hydrogen peroxide production was evaluated by DAB staining of leaf discs exposed to 5 mM FA for 72 h (Figure 2B). In all species, a brown precipitate was observed, consistent with H_2_O_2_ generation, and this precipitate was more pronounced in *P. americana* and *P. nigra*, while less precipitate was observed in the leaves of *C. sinensis* and *L. styraciflua*. Tissue cell death was assessed by electrolyte leakage and *P. nigra* exhibited the greatest increase in conductivity with a value of 52.8% (from 5.75 to 58.6%), followed by *P. americana* with 43.4% (from 0% to 43.4%), *L. styraciflua* with a 43.2% increase (from 12.04% to 55.10%), and, finally, *C. sinensis*, which exhibited the lowest electrolyte leakage of 24% (from 5.4% to 29.4%) (Figure 2C).

Because leaves turned brown and yellow after exposure to FA, the photosynthetic pigment content was quantified. Both chlorophyll (Figure 2D) and carotenoid (Figure 2E) contents were reduced after FA exposition in all species studied in this work. However, only *P. americana* exhibited significant differences, with a chlorophyll a and b content in control leaves of 2.08 ± 0.47 and 0.59 ± 0.13 mg g^−1^, respectively, which decreased to 1.23 ± 0.34 and 0.41 ± 0.08 mg g^−1^, respectively, representing a decrease of 40.9% of chlorophyll a and 30.3% of chlorophyll b. The carotenoid content also decreased significantly from 0.52 ± 0.09 mg g^−1^ in the control to 0.30 ± 0.06 mg g^−1^ in the FA-treated leaves, which represented a 42.5% decrease in carotenoid content.

Although the other species did not exhibit significant differences, decreases in the content of chlorophyll a, chlorophyll b, and carotenoids were observed. *L. styraciflua* exhibited decreases of 37.3%, 28.6%, and 34.5% in its content of chlorophyll a, chlorophyll b, and carotenoids, respectively. Leaves of *P. nigra* exhibited a 24.7%, 9.4%, and 25.2% decrease in their content of chlorophyll a, chlorophyll b, and carotenoids, respectively, and *C. sinensis* exhibited the lowest loss of photosynthetic pigments, with decreases of 11.3%, 13.1%, and 14.6% in its content of chlorophyll a, chlorophyll b, and carotenoids, respectively.

### 3.3. Fusaric Acid Toxicity Is Dependent of Its Foliar Content

**Figure 3:**
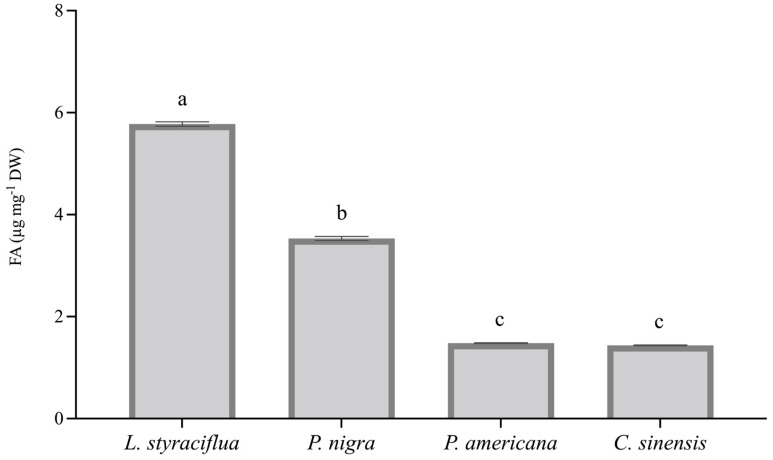
FA content in leaves of the four plant species after 72 h exposure to the toxin (FA 5 mM; 72 h). Data represent the mean of three samples ± standard deviation. Letters above the bars indicate significant differences as determined by ANOVA (*p* < 0.05) and Tukey test.

Because clear biochemical differences were observed in plant tissues treated with 5 mM FA, we quantified the toxin content in the leaves after 72 h exposure to determine the FA uptake by plant tissue. Figure 3 shows the content of FA in the leaves among species: *L. styraciflua* exhibited the highest FA content, followed by *P. nigra*, *P. americana*, and *C. sinensis*. The content of FA in the tissues correlated with the % of tissue cell death as assessed by electrolyte leakage.

### 3.4. FA Provoked Changes in the Specialized Metabolism in the Different Plant Species

**Figure 4:**
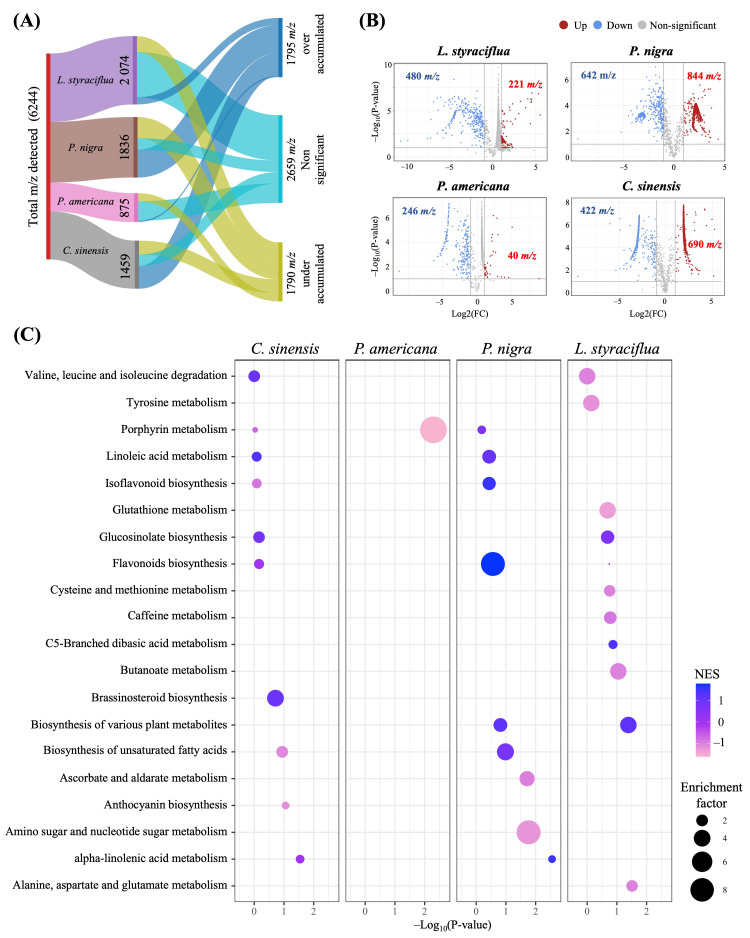
Analysis by untargeted metabolomics of the chemical response of plants after exposure to FA (5 mM). (**A**) Number of *m*/*z* ratios identified by the untargeted approach in leaves of each species studied. (**B**) Volcano plots comparing leaves exposed to FA and control treatments. (**C**) The top 20 metabolic pathways involved in the response to FA. Functional analysis was performed in MetaboAnalyst platform. NES: normalized enrichment score.

A chemical analysis was conducted to investigate the alterations in the leaves caused by the exposure to FA. Figure 4A depicts the *m*/*z* ratios detected by LC-HRMS. A total of 6244 *m*/*z* was observed in all the samples analyzed. Of these, 2074 *m*/*z* correspond to *L. styraciflua*, 1836 to *P. nigra*, and 1459 to *C. sinensis*, while 875 *m*/*z* were observed in *P. americana*. After performing comparative analyses in the MetaboAnalyst platform (Figure 4B), the *m*/*z* values that exhibited an intensity increase, a decrease, or non-significant changes in signal intensity following exposure to FA with respect to the leaves not exposed to the toxin were obtained. Most of the *m*/*z* ratio values (2659) did not exhibit significant changes in their intensities. However, the intensities of 1790 *m*/*z* decreased, while 1795 *m*/*z* increased, the content of FA. Of these, the latter is the most intriguing for further investigation.

Functional analysis employing the algorithms Mummichog and Metabolite Set Enrichment Analysis (MSEA) was performed using the differentially accumulated metabolites (DAMs) obtained in the comparative analyses. The 20 most enriched metabolic pathways are shown in Figure 4C, considering the enrichment factor of the Mummichog algorithm (circle size) and the Normalized Enrichment Score (NES) of the MSEA algorithm (color). This approach allows the identification of DAMs-enriched metabolic pathways after FA treatment. Only four metabolic pathways were identified as the most significant (*p* < 0.05) in at least one of the two algorithms employed: alpha-linolenic acid metabolism, flavonoid biosynthesis, isoflavonoid biosynthesis, and linoleic acid metabolism. Ten altered metabolic pathways were observed in *C. sinensis*, of which only linoleic acid metabolism, valine, leucine, and isoleucine catabolism, brassinosteroid biosynthesis, and glucosinolate biosynthesis showed an increase in FA-treated leaves. In *P. americana*, it is noteworthy that only the porphyrin metabolism pathway was altered, exhibiting a decrease in toxin-treated leaves. This result is associated with the quantification of photosynthetic pigments (Figure 2D). The species *P. nigra* exhibited the most intriguing alterations, with nine altered metabolic pathways identified. Notably, seven of these pathways exhibited increased accumulation in leaves exposed to FA. Finally, *L. styraciflua* exhibited the greatest number of altered metabolic pathways, with a total of 11. However, only the biosynthesis of various plant secondary metabolites, C5-branched dibasic acid metabolism, and glucosinolate biosynthesis pathway demonstrated an increase following FA stress. Once the major pathways that increase with FA exposure were identified, the 1795-increasing *m*/*z* ratios (Figure 4A) were used to search in databases and associate these values with the specific metabolites shown in Table 1. A total of 35 metabolites were tentatively identified, which were grouped into three categories according to their biosynthetic pathway. The most important category was phenylpropanoids, followed by fatty acids and, to a lesser extent, terpenoids. Among the phenylpropanoids, the flavonoids were the most notable. These included luteolin, apigenin, kaempferol, and naringenin, among others. The second group of metabolites was identified as unsaturated fatty acids, including palmitic, oleic, linoleic, and linolenic acids.

### 3.5. FA Alters the Content of Phenolic Compounds in Leaf Tissue

**Figure 5:**
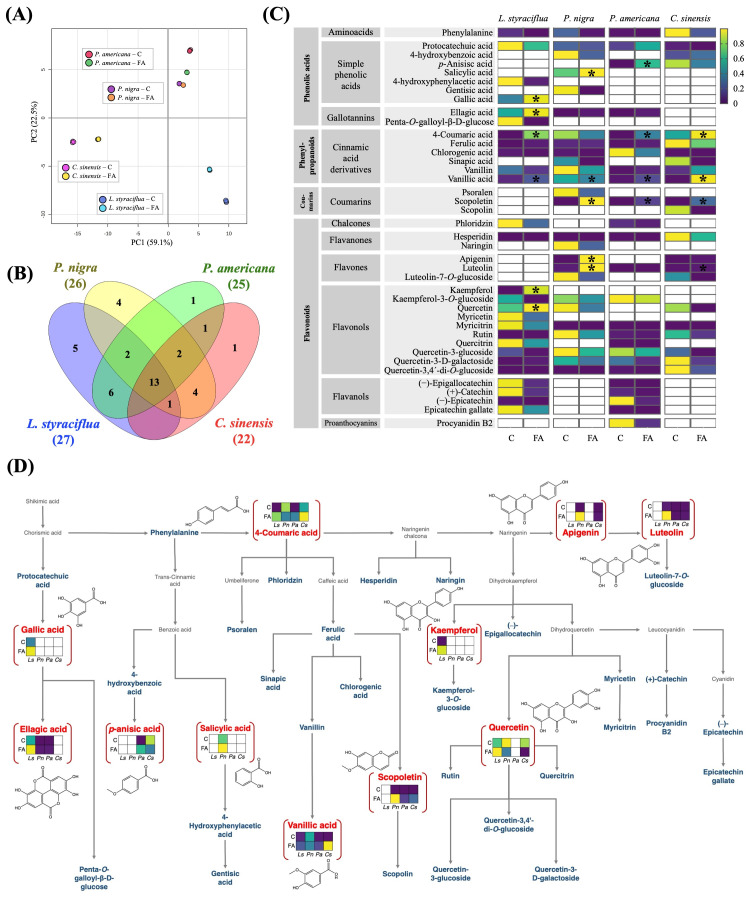
Targeted metabolomic analysis of phenolic compounds identified in the four plant species exposed to FA (5 mM). (**A**) Principal component analysis (PCA) showing similarities in phenolic composition between FA treatments and controls of the different species, statistical analysis was performed on MetaboAnalyst platform. (**B**) Venn diagram illustrating the number of phenolic compounds shared between the four study species. (**C**) Heat map of phenolic compounds identified and quantified in response to FA, normalization by Min-Max. * Indicates those metabolites that significantly increase their concentration after exposure to FA, statistical analysis was performed by *t*-tests (*p* < 0.05); (**D**) Approximation of the biosynthetic pathway of the 40 phenolic compounds detected in this work. The metabolites depicted in blue were detected in some species but exhibited a decrease in concentration. Conversely, the 11 metabolites illustrated in red exhibited an increase in at least one of the species studied. C: control (dH_2_O); FA: Fusaric acid; Ls: *L. styraciflua*; Pa: *P. americana*; Pn: *P. nigra*; Cs: *C. sinensis*.

Following the tentative identification of flavonoids, flavones, and flavonols as the most significantly altered metabolites following exposure to FA, the subsequent step involved the identity confirmation and quantification of molecules belonging to the phenolics pathway in leaves treated with the toxin relative to the control leaves. A total of 39 phenolic compounds plus the amino acid phenylalanine, which is a phenolic precursor, were identified and quantified in the four species under study. These included 27 in *L. styraciflua*, 26 in *P. nigra*, 25 in *P. americana*, and 22 in *C. sinensis* (Table S1). Figure 5A illustrates the PCA of the leaf extracts from the different treatments, with the phenolics content being the main separating factors, as well as the species and the exposure to FA or dH_2_O. This demonstrated how the toxin-treated leaf extracts were separated from the control leaves, thereby corroborating alterations in phenolic compound contents caused by the toxin. Figure 5B shows that 13 compounds were shared among all species (rutin, protocatechuic acid, quercetin-3,4′-di-O-glucoside, 4-coumaric acid, vanillic acid, quercetin-3-glucoside, chlorogenic acid, hesperidin, vanillin, ferulic acid, phenylalanine, quercetin-3-D-galactoside, and myricitrin). The phenolic compounds that were identified belong to different subgroups of metabolites. Most of them are flavonoids (21 metabolites) and phenolic acids (10 metabolites).

Most of the phenolic compounds identified exhibited a concentration decrease after FA treatment in comparison to control leaves (Figure 5C; Table S1). However, 11 metabolites were found at higher levels (Figure 5C,D): *p*-anisic acid, salicylic acid, gallic acid, ellagic acid, 4-coumaric acid, vanillic acid, scopoletin, apigenin, luteolin, kaempferol, and quercetin. Among the metabolites that exhibit increased concentrations following FA exposure, two particularly noteworthy molecules emerged: luteolin (flavone) and scopoletin (coumarin). These molecules increased their concentration in *C. sinensis*, *P. americana*, and *P. nigra* after FA exposure but they were not detected in the leaves of *L. styraciflua*.

### 3.6. Scopoletin Exerts a Protective Effect on the Leaves of Liquidambar Against FA Treatment

**Figure 6:**
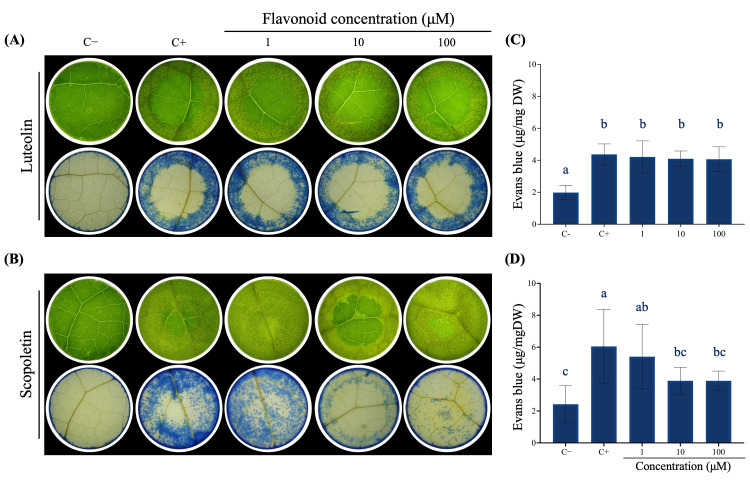
Effect of supplementing *L. styraciflua* leaves with flavonoids, followed by exposure to FA (2.5 mM). (**A**) Luteolin; foliar damage (up), tissue stained with Evans blue (down). (**B**) Scopoletin, foliar damage (up), tissue stained with Evans blue (down). (**C**,**D**) Amount of Evans blue dye. Different letters indicate statistically differences by ANOVA (*p* < 0.05) and Tukey test. C−: negative control (dH_2_O); C+: positive control (FA 2.5 mM).

To ascertain whether luteolin and scopoletin exert a protective effect against FA damage, leaves of *L. styraciflua*, the most FA-susceptible species, were exposed for 24 h to increasing concentrations of each flavonoid. Following this period, the leaves were exposed to FA (Figure 6). The protective effect of luteolin (Figure 6A and Figure S5) was not demonstrated, as no differences were observed in foliar damage nor in the amount of Evans blue dye that entered the cells (Figure 6C). This indicates that all leaves of *L. styraciflua* exhibited the same degree of cell death regardless of the luteolin concentration. On the other hand, when *L. styraciflua* leaves were exposed to increasing concentrations of scopoletin (Figure 6B and Figure S6), a slight decrease in leaf cell death was observed. In addition, significant differences were observed in the amount of Evans blue dye that entered the tissue, as well as at 10 and 100 mM of scopoletin (Figure 6D), which indicates that this coumarin induces modifications in plant tissues that allow them to overcome the stress generated by FA, and this effect is concentration-dependent.

## 4. Discussion

### 4.1. Selection of Woody Plants and Biochemical Disturbance After FA Exposure

Since its discovery in 1934 [57], the physiological effects of FA have been extensively studied in various *Fusarium* spp. plant hosts such as tomato, maize, cotton, tobacco, and banana. In this work, we focus on the study of woody species selected from a long list of host trees of *F. kuroshium* reported by [18], which included two forest species (*L. styraciflua* and *P. nigra*) and two crop species (*C. sinensis* and *P. americana*). In many diseases caused by *Fusarium* spp., the fungus penetrates through the roots. However, the group of ambrosial fungi (including *F. kuroshium*) that are vectored by ambrosial beetles can colonize through the xylem of plants. Under this scenario, leaf exposure to FA is more likely than diffusion with sap through the vascular tissues [1]. Previous studies have demonstrated that FA disrupts the photosynthetic machinery, causing oxidative bursts, altering plasma membranes, and finally inducing wilting and cell death [5,58,59,60]. In this work, we confirmed in woody plant leaves the biochemical and physiological changes previously observed in other species; the leaves treated with FA exhibited a notable reduction in biomass and a significant increase in electrolyte leakage, which can be attributed to the destruction of cell membranes, which leads to an increase in electrical conductivity. This rise in conductivity is an useful parameter for identifying the degree of cell death [61]. Our results showed that FA induced cell death; however, not all species studied presented the same damage degree, with *L. styraciflua* being the most susceptible and *C. sinensis* the most resistant, since, in *C. sinensis*, even in the highest concentration of FA, (10 mM) cell death was not observed. After quantifying the intracellular content of FA in the leaves, it was found that the entry of the toxin was four times lower in *C. sinensis* than in *L. styraciflua*; this may be related to physical factors or chemical aspects such as the production of antioxidant or antifungal metabolites. In this study, we addressed the chemical aspects. The presence of ROS was assessed by DAB staining, which allows the visualization of H_2_O_2_ content. Under normal conditions, ROS regulate processes such as senescence, phytoalexin production, photosynthesis, and the cell cycle. However, an imbalance in H_2_O_2_ content can induce oxidative stress, leading to the hypersensitive response (HR), a type of programmed cell death in response to pathogens [62,63,64]. In *Phaseolus vulgaris* L. plants infected with *F. oxysporum*, alterations in oxidative metabolism were observed in those that exhibited resistance to the pathogen. This highlights the role of ROS in the defense response [65]. In this study, it was observed that ROS generation was induced in all species 72 h after FA exposure, and this may be associated with the resistance of leaves to oxidative damage generated by the toxin. Nevertheless, it can also be linked to the induction of HR; the impact of FA on H_2_O_2_ production has been assessed in *Solanum lycopersicum* L., *Crocus sativus* L., and *Musa* sp., where the exposure to FA induces oxidative burst, which is caused by the production of O^2−^ and H_2_O_2_ as well as a decrease in activity of antioxidant enzymes. This outcome has been proposed as the principal mechanism of action of FA leading to the HR [1,10,60]. Under oxidative stress, the ultrastructure of chloroplasts may be affected, resulting in a reduction in chlorophyll content and photosynthetic activity [66]. This phenomenon has already been observed in *Lycopersicon esculentum* L. and *Gossypium hirsutum* plants infected by *F. oxysporum* and *Fusarium equiseti*, respectively. Following infection, damage to the photosynthetic machinery was observed, resulting from the production of ROS and membrane lipid peroxidation, which in turn led to a reduction in photosynthetic pigment concentrations and a decline in the photosynthetic rate [67,68]. Similarly, as observed in this study, exposure to FA resulted in a reduction in the photosynthetic pigment content in *Physalis peruviana*, *G. hirsutum*, and *Citrullus lanatus*. This pigment content and photosynthetic rate have been demonstrated to decrease as the toxin concentration and exposure time augment. It is proposed that the toxin could damage chloroplasts and decrease the performance of photosystems I and II, which may ultimately lead to energy disturbance and cell death [69,70,71,72].

### 4.2. Differential Metabolic Response Against FA in Woody Plants

FA can be recognized as an effector because it employs a variety of mechanisms to suppress immunity and alter or manipulate plant physiology, thereby facilitating the invasion and colonization of the pathogen [73]. Plants are capable of detecting effectors through the activation of effector-activated immunity (ETI), which elicits a series of metabolic changes to conduce the plant to an efficient defense response [74,75]. To gain insight into the metabolic response of plants to FA, we employed untargeted metabolomics. This analysis allowed us to comprehend the functions of certain specialized metabolites in response to stress generated by FA [76,77,78]. We employed a functional analysis, which allowed us to analyze the enrichment of metabolic pathways using metabolite libraries from databases. This approach facilitates the association of *m*/*z* values that show an increase or decrease in intensity with metabolic pathways that alter their accumulation in leaf tissue [79]. We identified the metabolism of linoleic and linolenic acid, both of which are unsaturated fatty acids that are abundant in plants and are integral components of membranes, cutin, and suberin in leaves [80]. Therefore, the increase in its concentration may be associated with structural damage to cell membranes generated by FA. Linolenic acid metabolism results in the formation of 12-OPDA, a molecule that has been tentatively identified in this work in three plant species, and this metabolism ultimately leads to the synthesis of jasmonates, a group of phytohormones that regulate the transcription of genes involved in responses to biotic and abiotic stress such as phytoalexin biosynthesis [81]. The two other most significant pathways were flavonoids and isoflavonoids metabolism. The metabolites of this family are distinguished by their high antioxidant capacity, which regulates the concentration of ROS in cells. In turn, they can act as chemical messengers, phytoalexins, or antimicrobial agents [82,83,84,85]. Similarly, this group of metabolites was the majority in the tentative identifications made in the four plant species of interest. Most of the metabolites belonging to this group were identified in *C. sinensis* and *P. nigra*, with seven and eight flavonoids being tentatively identified. This finding is consistent with the observation that both species are resistant to FA damage. In contrast, in *L. styraciflua*, the most susceptible species, only one flavonoid was tentatively identified. Flavonoids are a member of the large family of phenolic compounds [83]; therefore, in this work, by a targeted metabolomics approach using an in-house compounds library of approximately 60 standards of phenolic compounds, we identified 39 phenolics plus phenylalanine, the amino acid precursor, that were distributed in the four species studied. Most of the metabolites exhibited a reduction in concentration following exposure to FA, in accordance with the previously described physiological alterations generated by the toxin. Of particular interest were the 11 molecules that exhibited an increase in concentration (tannins, phenolic acids, phenylpropanoids, coumarin, flavones, and flavonols) after FA exposition, which suggest that the plant allocates its limited resources to the production of these metabolites as a defense mechanism against damage; *L. styraciflua*, the most susceptible species to FA, did not exhibit any of the flavones or coumarins detected in the other three species. Therefore, it was hypothesized that the supplementation of *L. styraciflua* leaves with some of these molecules will result in a reduction in the damage caused by FA.

### 4.3. Scopoletin Pretreatment Reduce FA-Induced Cell Death in L. styraciflua Leaves

Based on these results, luteolin and scopoletin were selected for evaluation, given that their concentrations were increased in two species that exhibited low susceptibility to damage and were not detected in *L. styraciflua*. This suggests that these molecules might play a role in counteracting the damage caused by the toxin. The results demonstrated that pretreatment of leaves with luteolin did not show a protective effect. Luteolin has been demonstrated to possess the capacity to impede the accumulation of trichothecene-type mycotoxins at the transcriptional level in *F. culmorum* and *F. graminearum* [86]. In addition to our findings, it is proposed that the leaves of *P. nigra* and *C. sinensis* may synthesize this phenolic compound in response to the detection of FA as a fungal toxin and attempt to inhibit the growth of the fungus. The second compound of interest was scopoletin, which was able to significantly reduce the cell death caused by FA in *L. styraciflua* leaves. This reduction indicates that scopoletin plays an important role in plants’ defense response against FA. Scopoletin has been identified as a phytoalexin, a molecule that is synthesized de novo in response to biotic or abiotic stress [87]. Shimizu and collaborators, identified the production of scopolin and its aglycone, scopoletin, in *Ipomoea purpurea* following exposure to *F. oxysporum* as a defense mechanism [88]. Also, scopoletin has been demonstrated to exhibit antifungal activity against *F. oxysporum* [89] and fungistatic activity against *Phakopsora pachyrhizi*, where it was associated with reduced ROS accumulation [90]. Although scopoletin and luteolin are the two molecules that increased their concentration in most of the resistant plants, it is important to note that the phenolic compounds may act in a synergistic manner and function as a collective antioxidant metabolite.

### 4.4. Proposed Model FA Action in Plants

**Figure 7:**
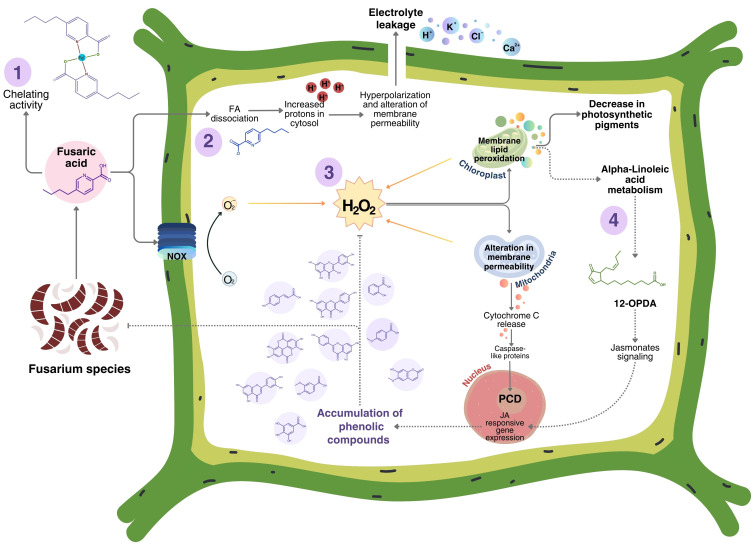
Model of the plant–FA interaction. Based on the available information and that obtained in this study, the interaction can be grouped into four key aspects. 1: FA can act indirectly against the plant by chelating micronutrients such as Zn^2+^, Mn^2+^, Cu^2+^, and Fe^2+^, which affects the plant’s resistance [11,91]. 2: Due to its weak acid character, FA can readily permeate cellular membranes, where it dissociates, releasing protons and acidifying the cytosol. This process activates the proton pump, induces depolarization, and alters the permeability of the membranes [8]. 3: FA causes an oxidative burst through NOX (NADPH oxidase), as well as in chloroplasts and mitochondria, resulting in damage to these organelles. In chloroplast membrane, the ROS accumulation leads to a decrease in the content of photosynthetic pigments, including carotenoids and chlorophyll a and b. In turn, the ROS increased mitochondrial membrane permeability, resulting in the release of cytochrome C. This initiates signal transduction through caspase-like proteins, leading to a hypersensitive response (HR), a type of programmed cell death (PCD) characterized by chromatin condensation and DNA degradation [9,10]. 4: In the final phase of the interaction and in response to FA stress, it is proposed that the toxin initiates signaling through jasmonates, which could induce the expression of biosynthetic genes for scopoletin, a primary antioxidant phytoalexin, providing effective protection against the elevated accumulation of ROS that are induced by FA within the various cellular organelles. Additionally, the biosynthesis of other phenolic compounds (tannins, phenolic acids, phenylpropanoids, coumarin, flavones, and flavonols) occurs, collectively reducing the elevated H_2_O_2_ content and inhibiting PCD. Some of these metabolites have been identified as antifungal compounds capable of impeding the growth of FA-producing fungi.

Based on the currently available information in model plants and the findings of this study in woody plants, we propose a model of the interaction between FA and host plants (Figure 7) [11,91]. This model is a guide for any type of plant, considering that each plant has its own characteristics. FA can act as a chelating agent, which results in a reduction in the availability of essential micronutrients for the plant. In addition, FA can induce cell death through signal transduction, acting on NADPH oxidase in cell membranes to generate H_2_O_2_, which increases the permeability of the mitochondrial membrane and releases cytochrome C into the cytosol. This, in turn, activates caspase-like proteins, leading to cell death [9,10,92]. In this study, Evans blue staining revealed the appearance of cell death in leaf tissue, which could be associated with a mechanism similar to that regulated by caspase-like proteins. Additionally, the loss of photosynthetic pigments, a reduction in biomass, and electrolyte leakage were noted. Following FA detection and accelerated ROS accumulation, signaling pathways are induced in plants, potentially through the jasmonate pathway, as evidenced by the tentative detection of the intermediate 12-OPDA in three species studied in this work. This leads to the activation of phytoalexin biosynthetic genes in response to toxin-generated stress. This is further supported by the findings of [93], which demonstrated that the biosynthesis of scopoletin and scopolin is regulated by jasmonate signaling through the activity of the transcription factor WRKY70; in addition, Sun and collaborators observed that jasmonate signaling was activated in *Nicotiana attenuata* following infection with *Alternaria alternata*, resulting in the accumulation of scopoletin [94]. It is not possible to exclude ethylene as part of the signaling pathway because it is already known that ethylene production is induced by FA exposure. For example, in tomato leaves infiltrated with a 1.4 mM FA solution, ethylene levels gradually increased, reaching a maximum (60%) at 12 h post-infiltration, and then decreased. Ethylene production preceded the generation of ROS and H_2_O_2_, as its maximum was reached at 48 h. However, O^2−^ showed a bimodal accumulation pattern, peaking at 4 h and then at 48 h post-infiltration. Ethylene is involved in modulating plant responses to biotic and abiotic agents and could enhance the oxidative burst [60]. Regarding salicylic acid (SA), there is not much evidence that SA is a key component of the defense response of plants against FA. Biochemical evidence such as SA accumulation upon FA treatment or genetic evidence as to whether *npr1 Arabidopsis* mutants are more tolerant to FA toxicity are still pending. In this study, SA was identified exclusively in *P. nigra*.

## 5. Conclusions

We found that fusaric acid generates different degrees of cell death in woody plants of ecological (*L. styraciflua* an *P. nigra*) and economical (*C. cinensis* and *P. americana*) importance that are potential hosts of *Fusarium* species such as *Fusarium kuroshium*, the causal agent of FD disease. Biochemical differences between the four tested plants were found, which contributes to the understanding of their susceptibility and response to this phytotoxin and provides insights into the complex plant–*Fusarium* interaction. Finally, scopoletin was identified as an important player in reducing FA-induced cell death.

## Figures and Tables

**Table 1:** Over-accumulated metabolites tentatively identified in FA-exposed leaf tissue.

Superclass	Class	Metabolite	*m* /*z*	R.t. (min)	Ionization Mode	Aduct	Mass Error (ppm)	Level ID *	Fragments	References
***C. sinensis***										
Flavonoids	Flavones	Schaftoside	565.1538	4.15	ESI+	[M+H]^+^	2.5	2	-	[24]
		Salvigenin	327.0870	7.79	ESI−	[M-H]^−^	1.2	1	311.1042; 297.0396	[25]
		Apigenin	269.0450	8.15	ESI−	[M-H]^−^	1.9	1	117.0331; 151.0031; 183.0432; 227.0329	[26]
		Chrysoeriol	301.0716	7.10	ESI+	[M+H]^+^	−3.0	1	286.0493; 258.0543; 153.0181	[27]
		Xanthomicrol	343.0818	8.73	ESI−	[M-H]^−^	1.5	1	300.0614	[28]
	Flavanones	Hesperetin	301.0710	5.94	ESI−	[M-H]^−^	2.7	1	286.0461; 242.0545; 164.0100; 151.0015; 134.0367	[29,30]
	Flavonols	Limocitrin	347.0760	5.18	ESI+	[M+H]^+^	0.3	1	332.0503; 301.0373	[31]
Triterpenoids	Limonoids	Limonexin	501.1759	6.00	ESI−	[M-H]^−^	1.4	1	457.1868; 455.1712; 429.1890	[32]
Apocarotenoids	Apocarotenoids-beta	Xanthoxin	249.1488	9.42	ESI−	[M-H]^−^	3.2	1	221.1171; 205.1595; 217.1605	[33]
Octadecanoids	Phytodienoic acids	12-Oxo-phytodienoic acid	293.2117	11.73	ESI+	[M+H]^+^	−2.0	2	-	[34]
Fatty acids and conjugates	Dicarboxylic acids	Azelaic acid	187.0972	5.02	ESI−	[M-H]^−^	2.1	1	125.0956; 97.0647; 123.0796	[35]
	Unsaturated fatty acids	Palmitic acid	255.2321	13.42	ESI−	[M-H]^−^	3.5	1	256.2352; 237.1833	[36,37]
		Linoleic acid	279.2322	13.55	ESI−	[M-H]^−^	2.9	1	277.2150	
		Oleic acid	281.2476	13.86	ESI−	[M-H]^−^	3.6	2	-	
***L. styraciflua***										
Phenolic acids	Gallotanins	1,2,3,4,6-pentagalloyl glucose	939.1089	4.53	ESI−	[M-H]^−^	2.1	1	787.0975; 769.0876; 617.0771; 169.0136	[38]
		Tellimagrandin II	937.0924	4.56	ESI−	[M-H]^−^	3.1	1	169.0143; 125.0230	[39]
Flavonoids	Flavonols	Quercetin-3-O-glucoside	463.0873	5.07	ESI−	[M-H]^−^	1.9	1	301.0718; 299.9899	[40]
Octadecanoids	Phyto- prostanes	F1-phytoprostane	327.2173	7.34	ESI−	[M-H]^−^	1.2	1	309.2061; 291.1953; 283.0642; 155.1073	[41,42]
	Phytodienoic acids	12-Oxo-phytodienoic acid	291.1958	10.27	ESI−	[M-H]^−^	2.7	1	273.1841; 255.0538; 207.1006	[43]
Fatty acids and conjugates	Unsaturated fatty acids	Linolenic acid	277.2163	12.69	ESI−	[M-H]^−^	3.6	1	233.2265	[44]
***P. nigra***										
Phenolic acids	Simple phenolic acids	Protocatechuic acid	153.0190	5.73	ESI−	[M-H]^−^	2.0	1	109.0291	[45]
Flavonoids	Flavones	Chrysin	253.0496	7.14	ESI−	[M-H]^−^	4.0	1	254.0533	[46]
		Genkwanin	283.0599	9.85	ESI−	[M-H]^−^	4.6	1	269.0416; 268.0356	[47]
		Luteolin	285.0396	6.13	ESI−	[M-H]^−^	3.2	1	283.0237; 151.0029; 133.0289; 107.0129; 175.0395; 151.0029; 133.0289; 107.0129	[48]
	Flavonols	Kaempferide	299.0552	7.22	ESI−	[M-H]^−^	3.0	1	284.0317; 227.0366; 107.0134	[49]
		Galangin	269.0444	7.30	ESI−	[M-H]^−^	4.1	1	239.129	[46]
	Flavanones	Naringenin	271.0602	7.13	ESI−	[M-H]^−^	3.7	1	273.0654; 272.0640	[48,50]
	Chalcones	Naringenin chalcone	253.0501	9.1	ESI−	[M-H_2_O-H]^−^	−0.1	1	151.0032; 107.0129	[51]
		Pinocembrin chalcone	255.0653	9.32	ESI−	[M-H]^−^	3.9	1	151.0030; 125.0963; 82.0122	[52]
	Dihydroflavonols	Aromadendrin	269.0446	6.90	ESI−	[M-H_2_O-H]^−^	1.5	1	151.0031; 193.0833; 151.0754; 125.0958; 121.0292; 93.0343	[47]
Apocarotenoids	Apocarotenoids-beta	Xanthoxin	249.1488	9.73	ESI−	[M-H]^−^	3.2	1	205.1596; 217.1223; 221.1163	[33]
Fatty acids and conjugates	Dicarboxylic acids	Azelaic acid	187.0969	5.27	ESI−	[M-H]^−^	3.7	1	125.0966; 97.0647	[53]
	Oxo fatty acids	Pyruvic acid	87.0084	0.50	ESI−	[M-H]^−^	4.6	2	-	[54]
	Unsaturated fatty acids	Stearic acid	283.2631	14.80	ESI−	[M-H]^−^	2.1	1	225.0580; 239.0731; 283.2639	
		Palmitic acid	255.2321	13.42	ESI−	[M-H]^−^	1.2	1	256.2358	[55]
***P. americana***										
Octadecanoids	Phytodienoic acids	12-Oxo-phytodienoic acid	291.1955	11.18	ESI−	[M-H]^−^	3.8	1	273.1844; 247.2053	[43,56]

* The level ID was established as: (1) indicates that there was a coincidence of the precursor ion and more than two fragment ions and (2) indicates those molecules where there was only a coincidence of the precursor ion, with a maximum mass error allowed of ± 5 ppm.

## Data Availability

The original contributions presented in this study are included in the article/Supplementary Material. Further inquiries can be directed to the corresponding authors.

## Supplementary Materials

The following supporting information can be downloaded at: https://www.mdpi.com/article/10.3390/jof11100745/s1, Figure S1: The effect of different concentrations of FA on leaf tissue of *L. styraciflua*; Figure S2: The effect of different concentrations of FA on leaf tissue of *P. nigra*; Figure S3: The effect of different concentrations of FA on leaf tissue of *P. americana*; Figure S4: The effect of different concentrations of FA on leaf tissue of *C. sinensis*; Figure S5: Effect of luteolin to the leaves of *L. styraciflua*, followed by exposure to FA (2.5 mM); Figure S6: Effect of scopoletin to the leaves of *L. styraciflua*, followed by exposure to FA (2.5 mM); Table S1: Phenolic compounds detected and quantified in leaves of plants expose to FA.
