# Supplementary material for: Phenolic Compounds Accumulation and Cell Death Degree Induced by Fusaric Acid in Agroforestry Hosts Plants of Fusarium Species
Source: J Fungi (Basel). 2025 Oct 17;11(10):745. doi: 10.3390/jof11100745 (PMC12565482; doi:10.3390/jof11100745)
Supplement: Supplementary file 1 [file jof-11-00745-s001.zip › jof-3892268-supplementary.pdf]

Supporting Information

Fig. S1 The effect of different concentrations of FA on leaf tissue of L. styraciflua. (a) Leaf damage. (b) Evans blue staining for cell death.

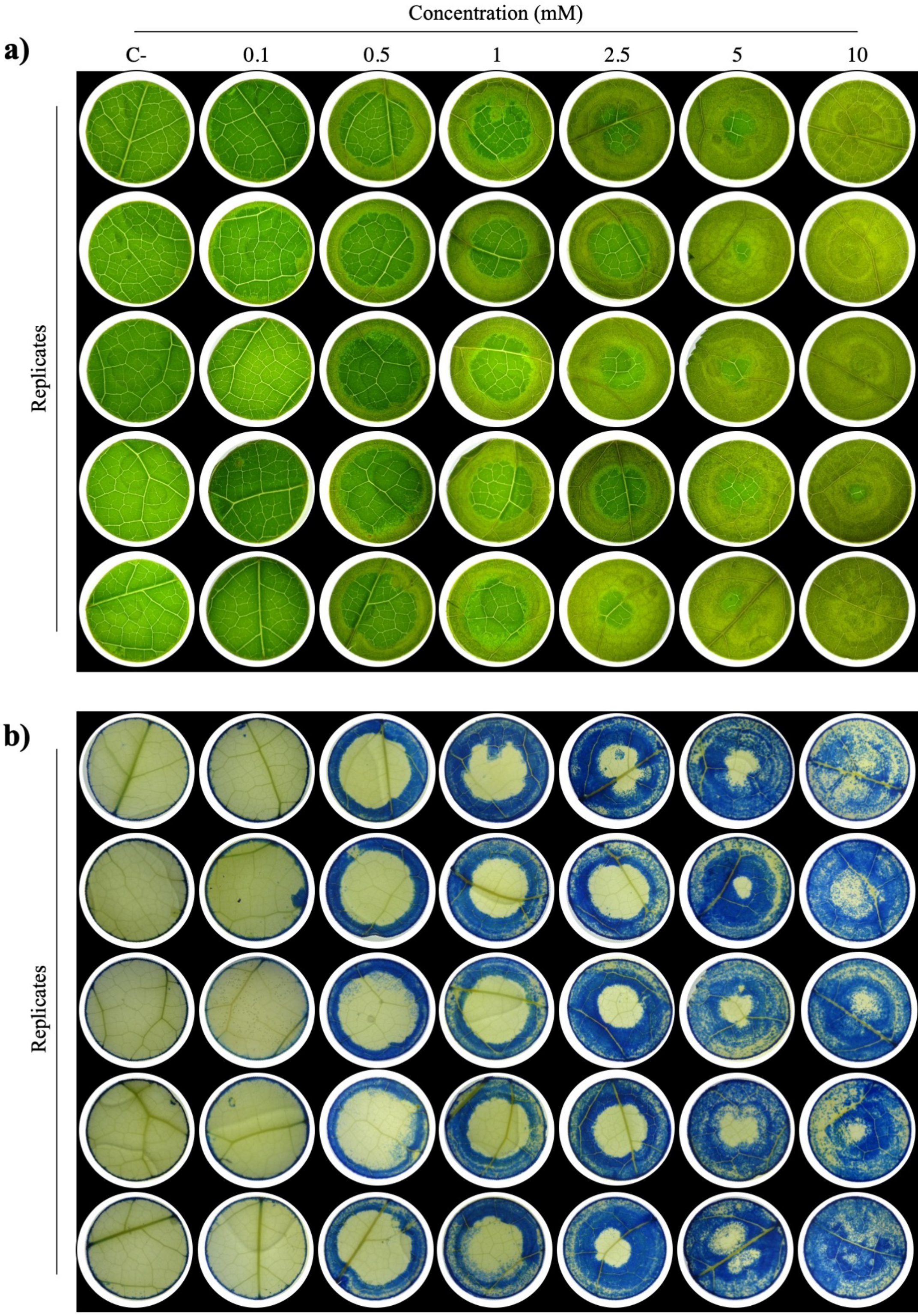

Fig. S2 The effect of different concentrations of FA on leaf tissue of P. nigra. (a) Leaf damage. (b) Evans blue staining for cell death.

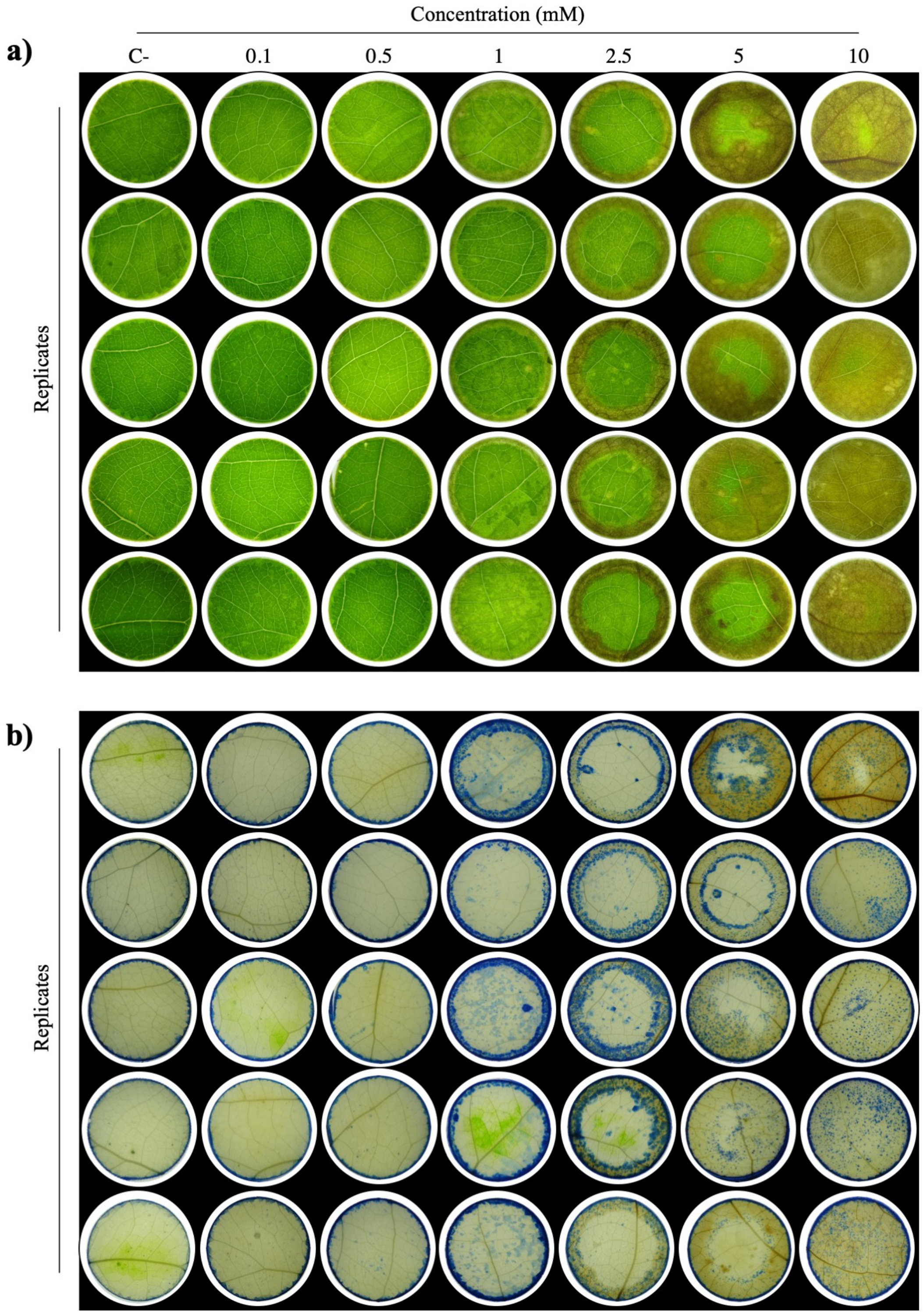

Fig. S3 The effect of different concentrations of FA on leaf tissue of P. americana. (a) Leaf damage. (b) Evans blue staining for cell death.

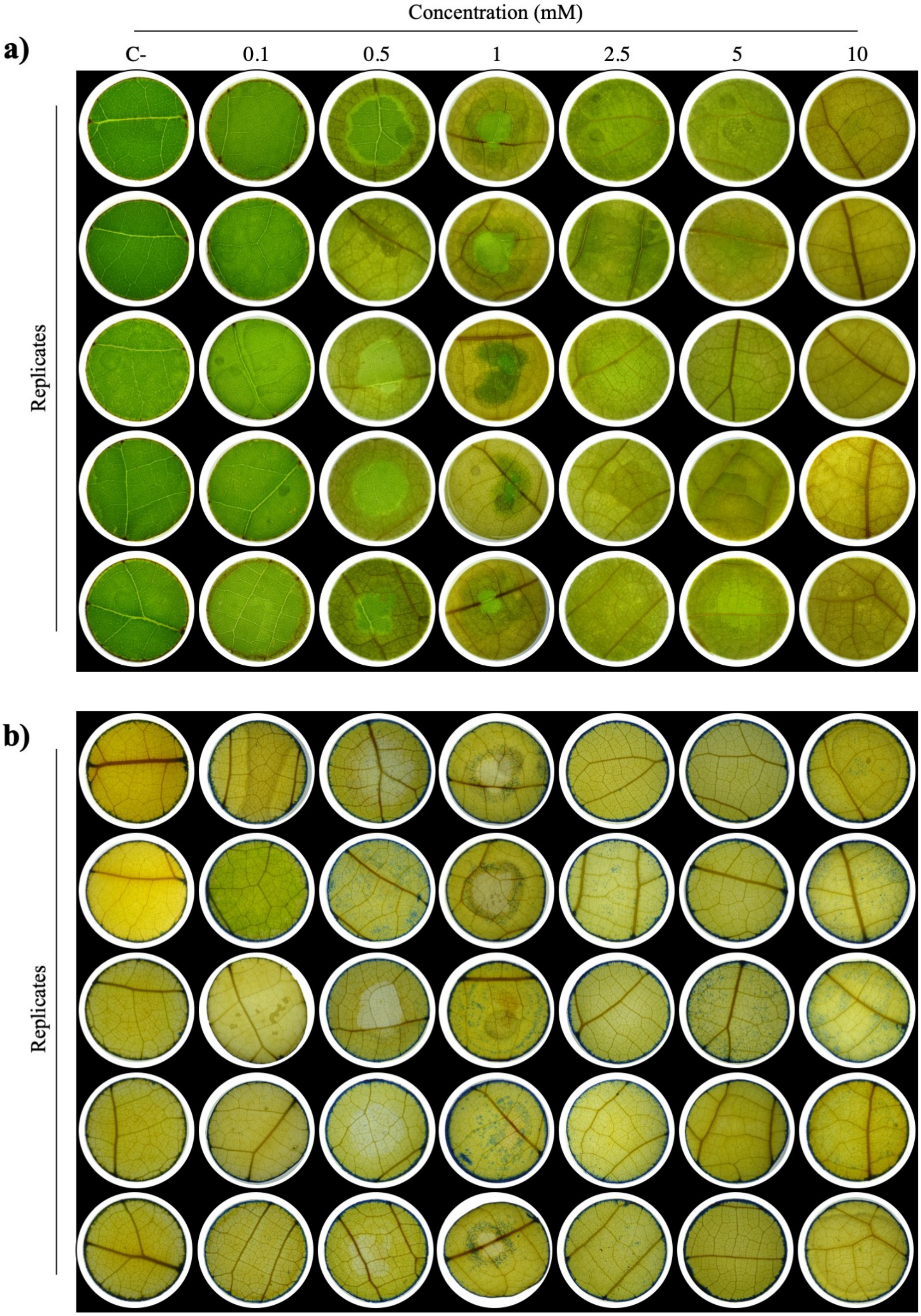

Fig. S4 The effect of different concentrations of FA on leaf tissue of C. sinensis. (a) Leaf damage. (b) Evans blue staining for cell death.

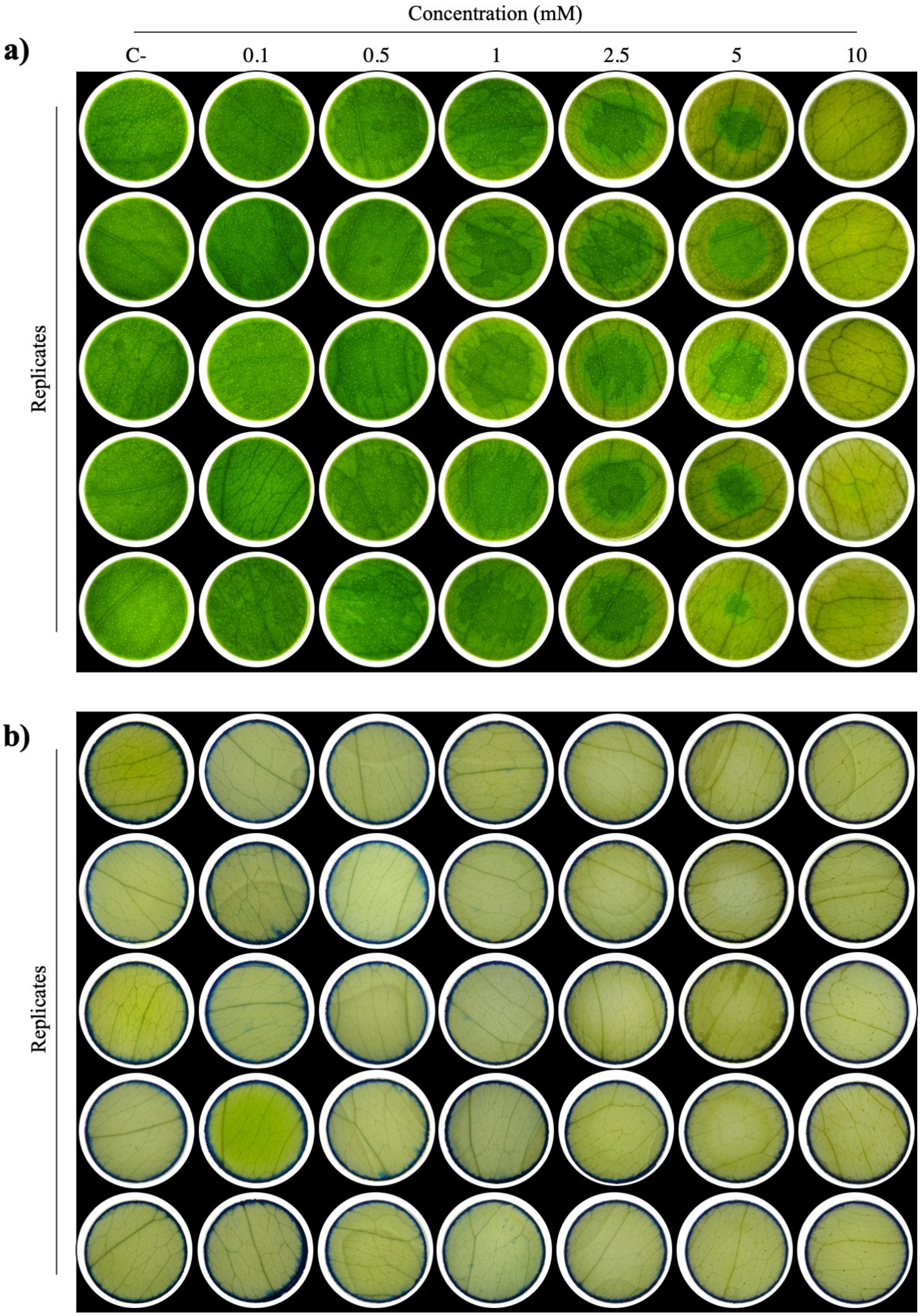

Fig. S5 Effect of luteolin to the leaves of L. styraciflua, followed by exposure to FA (2.5 mM). (a) Foliar damage (b) Tissue stained with Evans blue.

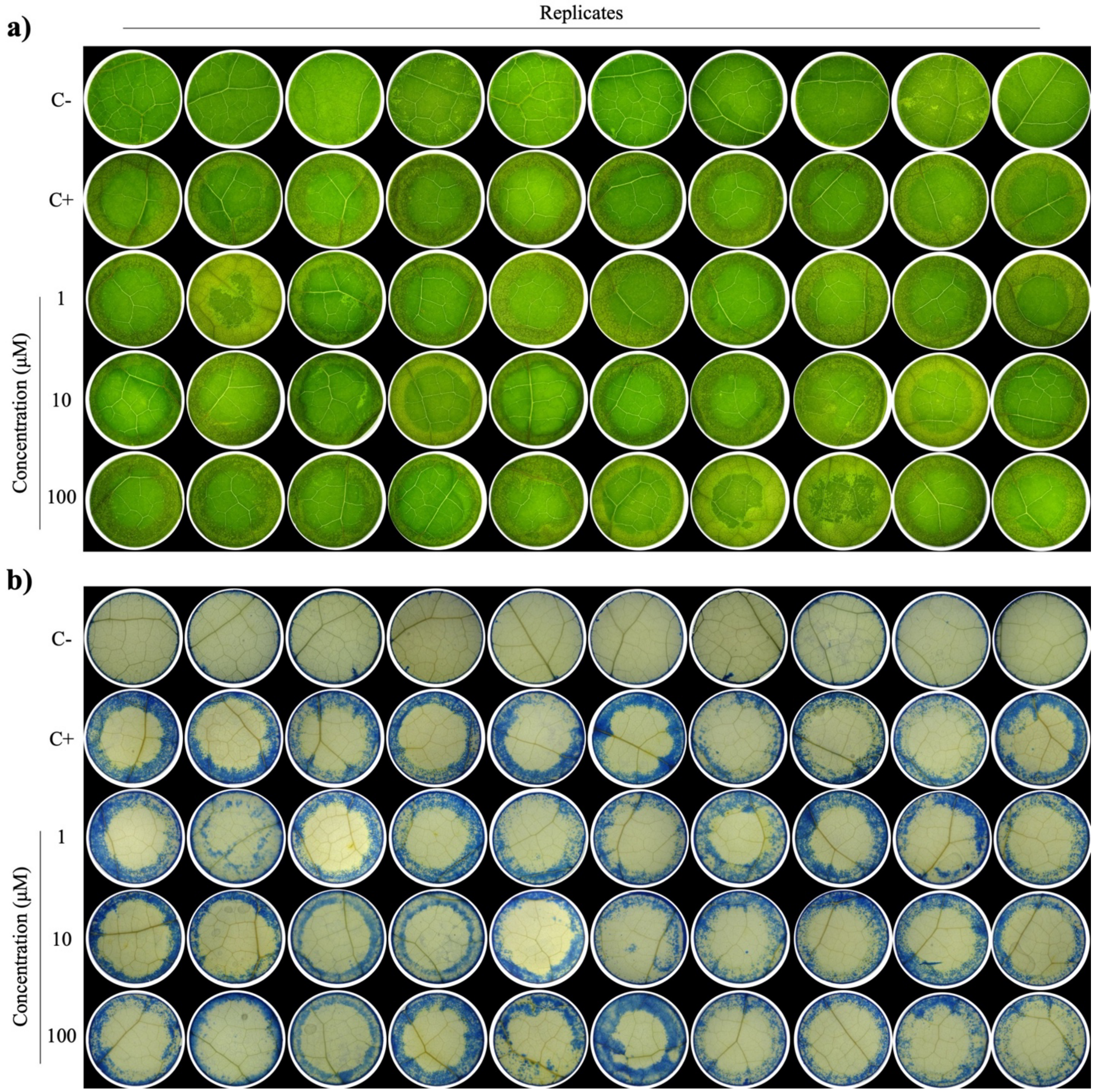

Fig. S6 Effect of scopoletin to the leaves of L. styraciflua, followed by exposure to FA (2.5 mM). (a) Foliar damage. (b) tissue stained with Evans blue.

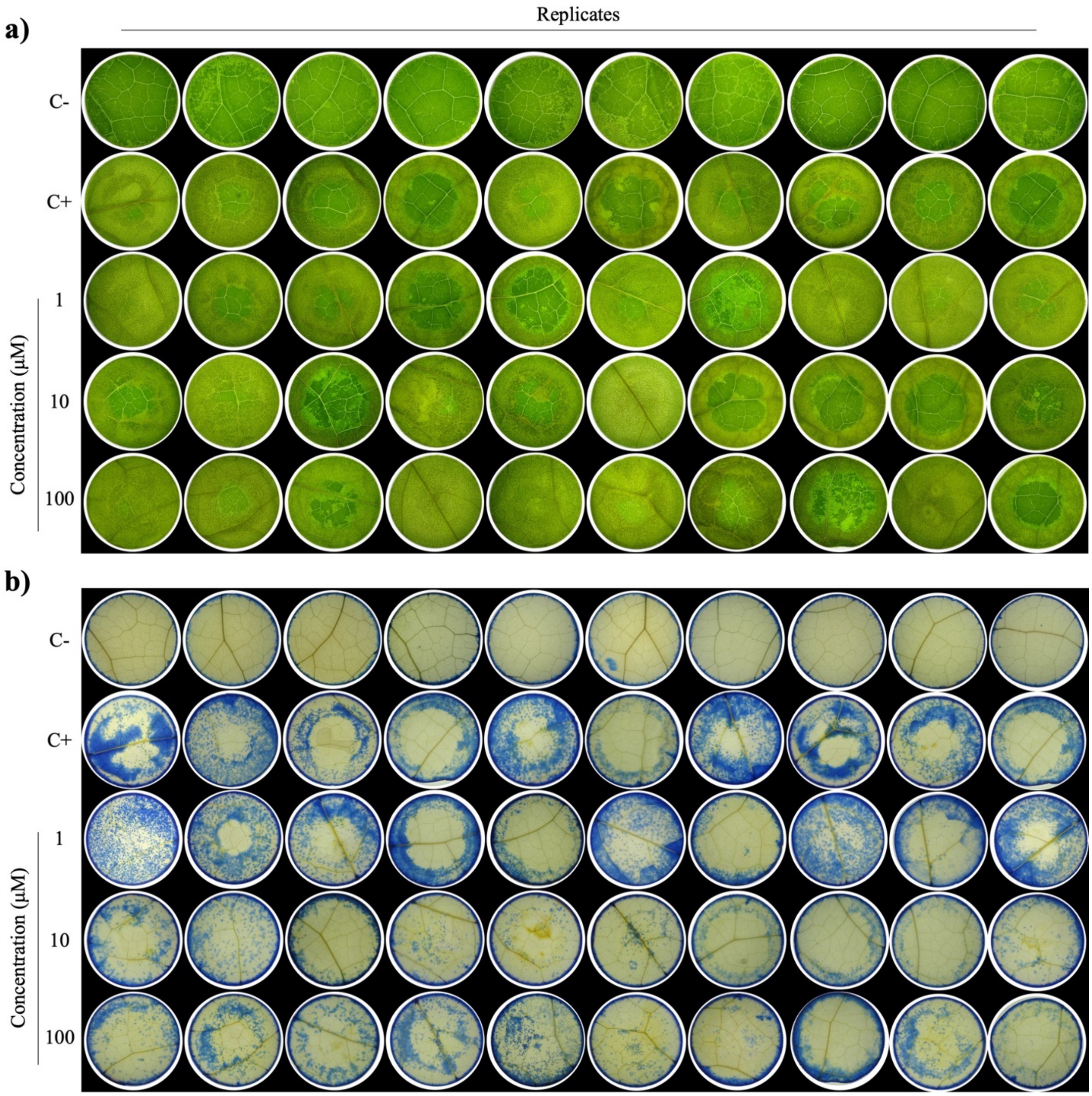

Table S1 Phenolic compounds detected and quantified in leaves of plants expose to FA

Species	*L. styraciflua*		*C. sinensis*		*P. americana*		*P. nigra*	
**Treatment**	**C**	**FA**	**C**	**FA**	**C**	**FA**	**C**	**FA**
Phenylalanine	19.44 ± 0.2	2.73 ± 0.0	201.82 ± 1.5	44.27 ± 0.1	5.00 ± 0.2	2.18 ± 0.0	53.30 ± 0.5	7.40 ± 0.0
Gallic acid	78.19 ± 0.6	205.77 ± 8.1	0.00 ± 0.0	0.00 ± 0.0	0.00 ± 0.0	0.00 ± 0.0	0.00 ± 0.0	0.00 ± 0.0
Protocatechuic acid	1.36 ± 0.0	0.90 ± 0.0	0.09 ± 0.0	0.03 ± 0.0	0.21 ± 0.0	0.77 ± 0.0	0.36 ± 0.0	0.30 ± 0.0
Gentisic acid	0.00 ± 0.0	0.00 ± 0.0	0.00 ± 0.0	0.00 ± 0.0	0.00 ± 0.0	0.00 ± 0.0	3.75 ± 0.1	0.00 ± 0.0
4-hydroxybenzoic acid	0.00 ± 0.0	0.00 ± 0.0	0.37 ± 0.0	0.59 ± 0.0	0.00 ± 0.0	0.00 ± 0.0	2.09 ± 0.0	0.49 ± 0.0
(-)-Epigallocatechin	47.67 ± 1.5	3.86 ± 0.2	0.00 ± 0.0	0.00 ± 0.0	0.37 ± 0.1	0.00 ± 0.0	0.00 ± 0.0	0.00 ± 0.0
4-hydroxyphenylacetic acid	30.31 ± 0.2	1.41 ± 0.2	0.00 ± 0.0	0.00 ± 0.0	0.00 ± 0.0	0.00 ± 0.0	0.00 ± 0.0	0.00 ± 0.0
(+)-Catechin	213.13 ± 4.5	19.14 ± 0.1	0.00 ± 0.0	0.00 ± 0.0	13.16 ± 0.1	2.9 ± 0.0	0.00 ± 0.0	0.00 ± 0.0
Scopolin	0.00 ± 0.0	0.00 ± 0.0	0.14 ± 0.0	0.00 ± 0.0	0.00 ± 0.0	0.00 ± 0.0	0.00 ± 0.0	0.00 ± 0.0
Vanillic acid	0.26 ± 0.0	0.46 ± 0.0	0.18 ± 0.0	1.49 ± 0.0	0.20 ± 0.0	0.39 ± 0.0	0.92 ± 0.0	0.68 ± 0.0
Myricitrin	1026.9 ± 40.3	421.49 ± 8	0.68 ± 0.2	0.00 ± 0.0	0.74 ± 0.1	0.00 ± 0.0	0.47 ± 0.0	0.00 ± 0.0
Salicylic acid	0.00 ± 0.0	0.00 ± 0.0	0.00 ± 0.0	0.00 ± 0.0	0.00 ± 0.0	0.00 ± 0.0	1.07 ± 0.0	1.46 ± 0.0
Ellagic acid	15.33 ± 1.0	26.96 ± 0.8	0.00 ± 0.0	0.00 ± 0.0	0.00 ± 0.0	1.04 ± 0.0	0.00 ± 0.0	0.77 ± 0.1
Rutin	0.64 ± 0.0	2.86 ± 0.0	271.7 ± 1.2	45.05 ± 0.8	16.35 ± 0.1	11.41 ± 0.1	427.19 ± 2.6	199.53 ± 0.6
Quercetin-3-D-galactoside	0.48 ± 0.0	0.86 ± 0.0	11.77 ± 0.1	1.75 ± 0.1	4.00 ± 0.1	1.37 ± 0.0	7.25 ± 0.1	4.00 ± 0.0
Quercetin-3-glucoside	2.22 ± 0.0	0.19 ± 0.0	3.36 ± 0.1	0.00 ± 0.0	10.72 ± 0.1	7.44 ± 0.1	12.36 ± 0.1	7.63 ± 0.0
Luteolin-7-*O*-glucoside	0.00 ± 0.0	0.00 ± 0.0	1.47 ± 0.1	0.00 ± 0.0	0.00 ± 0.0	0.00 ± 0.0	3.33 ± 0.0	0.76 ± 0.0
p-Anisic acid	0.00 ± 0.0	0.00 ± 0.0	0.62 ± 0.1	0.25 ± 0.0	0.00 ± 0.0	0.45 ± 0.0	0.00 ± 0.0	0.00 ± 0.0
Penta-*O*-galloyl-B-D-glucose	1056.25 ± 14.8	0.00 ± 0.0	0.00 ± 0.0	0.00 ± 0.0	0.00 ± 0.0	0.00 ± 0.0	0.00 ± 0.0	0.00 ± 0.0
Naringin	0.00 ± 0.0	0.00 ± 0.0	0.00 ± 0.0	0.00 ± 0.0	0.00 ± 0.0	0.00 ± 0.0	34.3 ± 0.2	8.39 ± 0.2
Chlorogenic acid	36.91 ± 0.1	12.31 ± 0.1	6.18 ± 0.2	0.23 ± 0.0	1215.01 ± 28.1	444.66 ± 0.8	30.84 ± 0.6	9.13 ± 0.0
Procyanidin B2	0.00 ± 0.0	0.00 ± 0.0	0.00 ± 0.0	0.00 ± 0.0	500.65 ± 7.1	46.56 ± 0.4	0.00 ± 0.0	0.00 ± 0.0
(-)-Epicatechin	5.88 ± 0.0	0.00 ± 0.0	0.00 ± 0.0	0.00 ± 0.0	315.99 ± 2.6	16.87 ± 0.2	0.00 ± 0.0	0.00 ± 0.0
Vanillin	2.36 ± 0.1	1.18 ± 0.0	1.04 ± 0.0	3.62 ± 0.0	0.58 ± 0.0	0.91 ± 0.0	6.56 ± 0.0	3.51 ± 0.0
4-Coumaric acid	0.00 ± 0.0	1.31 ± 0.0	0.87 ± 0.0	1.59 ± 0.0	0.00 ± 0.0	0.6 ± 0.0	1.36 ± 0.0	0.6 ± 0.0

Species	*L. styraciflua*		*C. sinensis*		*P. americana*		*P. nigra*	
**Treatment**	**C**	**FA**	**C**	**FA**	**C**	**FA**	C	**FA**
Quercetin-3,4'-di-*O*-glucoside	0.00 ± 0.0	0.28 ± 0.0	833.91 ± 3.7	167.75 ± 1.8	18.29 ± 0.6	7.1 ± 0.1	0.56 ± 0.0	0.00 ± 0.0
Scopoletin	0.00 ± 0.0	0.00 ± 0.0	0.00 ± 0.0	2.68 ± 0.0	0.00 ± 0.0	1.35 ± 0.1	0.00 ± 0.0	9.86 ± 0.0
Ferulic acid	0.00 ± 0.0	0.13 ± 0.0	3.93 ± 0.0	2.99 ± 0	0.03 ± 0.0	0.16 ± 0.0	0.25 ± 0.0	0.28 ± 0.0
Sinapic acid	0.00 ± 0.0	0.00 ± 0.0	0.59 ± 0.1	0.00 ± 0.0	0.00 ± 0.0	0.00 ± 0.0	0.3 ± 0.0	0.00 ± 0.0
Epicatechin gallate	148.24 ± 4.6	66.77 ± 1.6	0.00 ± 0.0	0.00 ± 0.0	0.46 ± 0.0	0.00 ± 0.0	0.00 ± 0.0	0.00 ± 0.0
Quercitrin	83.94 ± 0.8	40.27 ± 0.2	0.00 ± 0.0	0.00 ± 0.0	0.09 ± 0.0	0.01 ± 0.0	0.00 ± 0.0	0.00 ± 0.0
Kaempferol-3-*O*-glucoside	2.17 ± 0.0	0.00 ± 0.0	0.00 ± 0.0	0.00 ± 0.0	3.79 ± 0.1	3.44 ± 0.0	3.08 ± 0.0	1.73 ± 0.0
Hesperidin	0.00 ± 0.0	4.48 ± 0.1	7403.7 ± 119.8	4572.93 ± 64.9	31.52 ± 2.7	18.93 ± 0.1	9.52 ± 0.3	5.94 ± 0.1
Myricetin	37.2 ± 1.0	10.66 ± 0.2	0.00 ± 0.0	0.00 ± 0.0	0.00 ± 0.0	0.00 ± 0.0	0.00 ± 0.0	0.00 ± 0.0
Phloridzin	47.57 ± 0.0	12.75 ± 0.1	0.00 ± 0.0	0.00 ± 0.0	3.62 ± 0.0	1.92 ± 0.0	0.00 ± 0.0	0.00 ± 0.0
Quercetin	1.35 ± 0.0	1.92 ± 0.0	1.64 ± 0.0	0.00 ± 0.0	0.00 ± 0.0	0.00 ± 0.0	1.91 ± 0.0	0.64 ± 0.0
Luteolin	0.00 ± 0.0	0.00 ± 0.0	0.74 ± 0.0	1.95 ± 0.1	0.00 ± 0.0	0.42 ± 0.0	2.67 ± 0.0	74.22 ± 0.5
Psoralen	0.00 ± 0.0	0.00 ± 0.0	0.00 ± 0.0	0.00 ± 0.0	0.00 ± 0.0	0.00 ± 0.0	0.22 ± 0.0	0.06 ± 0.0
Apigenin	0.00 ± 0.0	0.00 ± 0.0	0.00 ± 0.0	0.37 ± 0.0	0.00 ± 0.0	0.00 ± 0.0	0.00 ± 0.0	15.95 ± 0.1
Kaempferol	0.00 ± 0.0	0.79 ± 0.1	0.00 ± 0.0	0.00 ± 0.0	0.00 ± 0.0	0.00 ± 0.0	0.00 ± 0.0	0.00 ± 0.0

Data are expressed as mean ± standard deviation (n=3) reported in µg/g of dried leaves. C: Control; FA: Fusaric acid
